# Unmet Needs and Quality of Life of Cancer Patients and Their Families: Actor–Partner Interdependence Modeling

**DOI:** 10.3390/healthcare9070874

**Published:** 2021-07-12

**Authors:** Yubeen Jang, Younhee Jeong

**Affiliations:** College of Nursing Science, Kyung Hee University, Seoul 02447, Korea; yubeen@khu.ac.kr

**Keywords:** quality of life, cancer patients, family, unmet needs, dyadic analysis

## Abstract

Unmet needs and quality of life (QOL) are important nursing issues for both patients and their families. However, studies into their direct association, considering the dyadic relationship between them, have not been done. We investigated the associations using the actor–partner interdependence modeling for dyadic data. Data were collected from 115 patient–family dyads at a tertiary teaching hospital. The study variables were assessed using the questionnaires and clinical data. To analyze patient–family dyad data, the actor–partner interdependence modeling and structural equation modeling were used. The cancer patients and their families experienced diverse and high levels of unmet needs that affected their quality of life, both physically and mentally. The cancer patients’ unmet needs decreased their physical and mental quality of life, while those of their families had a negative impact on their own physical and mental quality of life. However, the cancer patients’ unmet needs did not have partner effects on their families’ quality of life, and vice versa. Therefore, unmet needs played important roles in their QOL taking into dyadic relationships in the model. The results suggest that nursing intervention programs to meet the needs of both patients and their families are required to improve their quality of life.

## 1. Introduction

Cancer treatment has focused on the cure itself, especially on solving physical problems, in the past. However, in recent years, multi-dimensional perspectives, including physical and psychological quality of life (QOL), have been considered important aspects of cancer management [1,2]. Cancer is a traumatic experience that is a reminder of death to the patients, and their families experience the burden of care and the fear of losing their loved ones [3]. When family members have serious or chronic diseases, it causes impairment of family functioning and in turn affects the treatment outcomes of the patient negatively; thus, care is necessary considering the QOL of both cancer patients and their families [4].

The cancer patients’ symptom experiences, including pain, depression, psychological distress, and uncertainty, influence the patient’s own QOL and the QOL of their families [5,6]. The burden of care, resilience, and the degree of social support of the families are also known to affect the QOL of both cancer patients and their families [7]. In addition, QOL differs depending on the degree of needs met [8]. Cancer patients and their families have needs as they experience confusion and unprecedented feelings about unwanted situations, such as a cancer diagnosis [9].

Unmet needs are unresolved demands that help to determine the priority of nursing intervention by reflecting the most urgent requirements of cancer patients and their families [10]. Nevertheless, the patients and their families tend to be reluctant to express their psychosocial problems or discomforts because they regard those problems as inevitable issues caused by the illness [11]. Consequently, accurate identification of needs is more difficult [12]. As the measurement tools for unmet needs have been developed recently, the research on unmet needs of both cancer patients and their informal caregivers, including their families, has become more active [13]. The studies have shown that cancer patients and their families have various domains and degrees of unmet needs, and perceptions of unmet needs between cancer patients and their physicians not always consistent [13,14]. Therefore, it is important to properly assess and address the unmet needs of both.

In addition to evaluate the unmet needs, their effects on QOL of both patients and their families should be studied. There are studies assessing QOL and unmet needs of cancer patients or family caregivers. A study with breast cancer survivors showed that the survivors had high unmet needs and lower QOL [8]. However, the study did not investigate the relationship between unmet needs and QOL. The other study with the patients with colorectal cancer showed that unmet needs were negatively associated with QOL [15]. The study with family caregivers of cancer patients presented a negative correlation between unmet needs and QOL [16].

Most quantitative studies on the association between unmet needs and QOL of cancer patients and their families have been conducted with parametric analyses. Because the two share common backgrounds and have a significant impact on each other, the dyadic data violate the assumption of independence of observations in parametric statistical analysis. Therefore, interdependence as a dyad should be considered when studying patients and their families at the same time, and appropriate analysis must be performed by including non-independence in the model [17]. The actor–partner interdependence modeling (APIM) approach has been considered as a proper method to test interdependence in dyad data [18]. However, the APIM approach on the impact of unmet needs on the QOL of cancer patients and their families has not been conducted thus far. Therefore, we investigated the impact of unmet needs of cancer patients and their families on the QOL in the dyadic context using the APIM approach in this study.

## 2. Methods

### 2.1. Hypothetical Models

**Figure 1:**
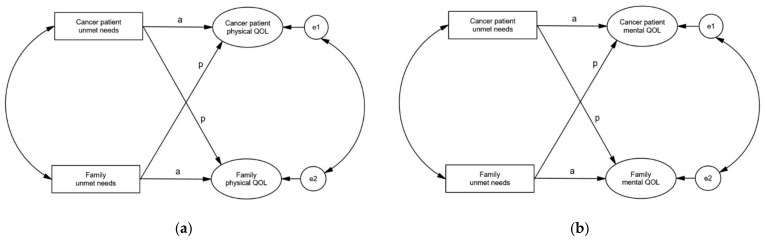
Hypothetical models. (**a**) Physical QOL; (**b**) Mental QOL. Note. a: actor effect; p: partner effect.

A hypothetical model was constructed by reviewing literature and reflecting interdependence using APIM as shown in Figure 1. In this model, unmet needs were predictor variables and physical and mental QOL were response variables. Taking into the characteristics of dyadic data, both actor effects and partner effects were tested in the model. Actor effects are predictor variable’s effects on own response variable. Partner effects are predictor variable’s effects on the response variable of the counterpart. The hypotheses of this study were the following:

#### 2.1.1. Actor Effects

- Cancer patients’ unmet needs would be associated with cancer patients’ physical QOL.
- Cancer patients’ unmet needs would be associated with cancer patients’ mental QOL.
- The unmet needs of the families of cancer patients would be associated with the physical QOL of the families.
- The unmet needs of the families of cancer patients would be associated with the mental QOL of the families.

#### 2.1.2. Partner Effects

- Cancer patients’ unmet needs would be associated with their families’ physical QOL.
- Cancer patients’ unmet needs would be associated with their families’ mental QOL.
- The unmet needs of the families would be associated with the physical QOL of the patients.
- The unmet needs of the families would be associated with the mental QOL of the patients.

### 2.2. Design and Sample

The data for this cross-sectional descriptive study were collected from patient–family dyads at a tertiary teaching hospital from 15 April to 24 September 2020. The patient participants were those who had been diagnosed with cancer and were undergoing inpatient treatments. Family member participants were the main adult members caring for cancer patients. If either of them disagreed to participate, they were excluded from the study. A minimum sample size for structural equation modeling is recommended from 50 to 200 depending on complexity of the model [19,20]. We recruited 119 patient–family dyads, but two dyads opted out due to deterioration of the patient condition. The data from two dyads were excluded due to incomplete responses. A total of 115 dyads were included in the final analysis.

### 2.3. Ethical Approval

This research was approved by the Institutional Review Board (IRB). All study procedures performed were in accordance with the ethical standards of the IRB and the 1964 Helsinki declaration, as well as its later amendments or comparable ethical standards. Informed consent was obtained from all patients and family members who participated in the study.

### 2.4. Measurement

The self-report questionnaires consisted of questions regarding the participants’ demographic and disease-related characteristics, unmet needs, and QOL. The general characteristics included demographic features, such as gender, age, religion, employment status, monthly income, and education level. The cancer-related characteristics were assessed through the electronic health record. Furthermore, cancer diagnosis, the Karnofsky Performance Status (KPS), treatment modality, recurrence, and metastasis were examined. To reduce response bias, participants responded individually to the questionnaire while the researchers were not present.

#### 2.4.1. Unmet Needs

The cancer patient’s unmet needs were assessed using the Comprehensive Needs Assessment Tool in Cancer (CNAT) developed by Shim, et al. [21]. For assessing the needs of the family, the Comprehensive Needs Assessment Tool for Cancer-Caregivers (CNAT-C) developed by Shin, et al. [22] was used. The higher the score, the greater the unmet needs. In this study, the Cronbach’s alphas of the CNAT and the CNAT-C were 0.89 and 0.86, respectively.

#### 2.4.2. QOL

QOL was measured using the 36-item Short-Form Health Survey (SF-36v2 ^®^; QualityMetric Incorporated, Lincoln, RI, USA) that consists of eight health domains including physical functioning, role limitation due to physical health, bodily pain, general health, vitality, social functioning, role limitation due to emotional problems, and mental health. The Physical Component Summary (PCS) and the Mental Component Summary (MCS) are composites of the weighted domain scales representing physical and mental QOL, respectively. Higher scores in PCS and MCS are indicative of greater physical and mental QOL. In this study, the Cronbach’s alpha reliability of this measure was 0.89.

### 2.5. Data Analysis

IBM Statistical Package for the Social Sciences (SPSS Statistics) version 25.0 and AMOS 22.0 (IBM, Chicago, IL, USA) were used for statistical analysis. Data were summarized using means and standard deviations (SD). Unmet needs and QOL differences based on the general and disease-related characteristics were analyzed using independent *t*-tests and one-way analysis of variance (ANOVA). The relationship between unmet needs and QOL was analyzed by the Pearson’s correlation coefficient. To examine the patient–family dyad data by the APIM, structural equation modeling was used. Prior to conducting the APIM, the following assumptions were tested: normality with skewness and kurtosis. The correlations between the measured variables were analyzed using the Pearson’s correlation coefficient. To verify the dyadic modeling, *χ*^2^, *χ*^2^/df, Standard Root Mean Squared Residual (SRMR), Goodness of Fit Index (GFI), Normed Fit Index (NFI), Tucker-Lewis Index (TLI), Comparative Normed of Fit Index (CFI), and Root Mean Square Error of Approximation (RMSEA) were utilized.

## 3. Results

The characteristics of the participants are presented in Table 1. In summary, patient participants included 65 men (56.5%) and 50 women (43.5%), with a mean age of approximately 62 years. Most participants were unemployed (79.1%). The most frequent cancer diagnoses were lymphoma (20.9%) and breast cancer (18.3%). Most patient respondents were undergoing chemotherapy (72.2%), and had metastatic cancers (84.3%). The functional performance based on KPS demonstrated that 59% of had no functional impairment. The family participants comprised 30 men (26.1%) and 85 women (73.9%), with a mean age of approximately 51 years. Most of them were living with the patients (72.2%), and about a half of them (54.8%) were employed.

The descriptive statistics of unmet needs and QOL are shown in Table 2. The patient’s mean CNAT score was 50.93 (SD = 19.56), and the families’ mean CNAT-C score was 46.52 (SD = 20.68). The domains with higher unmet needs for the patients were those related to healthcare staff (66.99 ± 27.80), information (66.35 ± 21.81), and psychological problems (59.01 ± 25.97). As compared to the patients, the domains of the greater unmet needs for the families were healthcare staff (67.83 ± 27.73), information (62.36 ± 24.73), and hospital facilities and services (49.28 ± 26.64). These differences were not significant based on their general characteristics, excluding employment status (Table 3). The unemployed patients (53.77 ± 18.70) had higher unmet needs than those who were employed (40.17 ± 19.39; *p* = 0.002).

The PCS scores for the patients and families were 38.06 (SD = 8.27) and 49.38 (SD = 7.68), respectively (Table 2). The patients’ PCS differed significantly depending on the employment status (unemployed vs. employed; 36.96 ± 8.36 vs. 42.22 ± 6.52, respectively; *p* = 0.005; Table 3). Among cancer-related characteristics, treatment modality and KPS scores were associated with PCS scores. The patients who received chemotherapy had higher PCS scores than those who were received radiation therapy (40.00 ± 7.22 and 26.32 ± 8.32, respectively; *p* < 0.001). Those with a KPS of 80 or above had a higher PCS score than those with a KPS of less than 40 (41.31 ± 6.84 and 27.38 ± 4.41, respectively; *p* < 0.001). The families’ PCS differed significantly depending on the employment status (unemployed vs. employed; 47.58 ± 7.86 vs. 50.86 ± 7.27, respectively; *p* = 0.022), and alternative informal caregiver (having alternative caregiver vs. no alternative caregiver; 51.84 ± 7.09 vs. 45.94 ± 7.21; respectively; *p* = < 0.001). In addition, the younger the family members, the higher the PCS scores.

The MCS scores were 37.93 (SD = 11.24) and 42.13 (SD = 11.47) for the patients and families, respectively. The patients’ MCS differed significantly depending on the employment status (unemployed vs. employed; 36.81 ± 11.24 vs. 42.20 ± 10.37, respectively; *p* = 0.036; Table 3). The patients’ MCS scores depended on their KPS scores rather than the general characteristics; those with a KPS of 80 or above had a higher MCS score than those with a KPS of less than 40 (40.41 ± 11.95 and 30.12 ± 6.98, respectively; *p* = 0.010; Table 3). The families’ MCS differed significantly, depending on the employment status (unemployed vs. employed; 39.23 ± 11.55 vs. 44.53 ± 10.91, respectively; *p* = 0.013; Table 4) and living with the patient (living with vs. without; 40.76 ± 11.73 vs. 45.68 ± 10.06; respectively; *p* = 0.039).

Because the absolute values of skewness and kurtosis of the measured variables were less than 2 (Table 2), the normality assumption was met. Additionally, according to Mardia’s multivariate normality test, the multivariate normality was met as well (0.166, *p* > 0.05). Therefore, the maximal likelihood estimation was used for the analysis. The correlation between the variables of the cancer patients and their families is presented in Table 5. The absolute values of correlation coefficients were between 0.03 and 0.39, thus confirming the absence of a multicollinearity issue.

**Figure 2:**
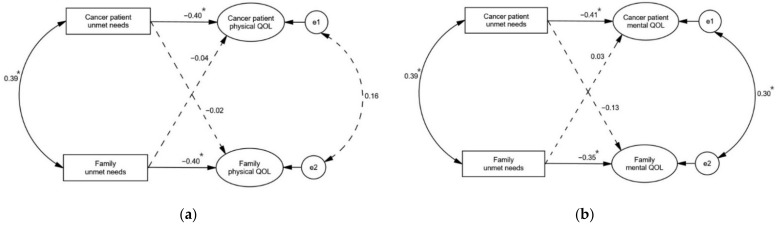
Final models. (**a**) Physical QOL; (**b**) Mental QOL. Note. Dashed arrows represent statistical non−significance; * *p* < 0.05.

The APIM was used to determine the dyadic effects of unmet needs on physical (PCS) and mental QOL (MCS). All indices were suggestive of good fit of the models (Table 6 and Table 7). Consistent with the hypothetical models, a significant actor effect of unmet needs was observed on PCS for both the cancer patients (β = −0.396, *p* < 0.001; Table 6 and Figure 2) and their families (β = −0.403, *p* < 0.001; Table 6 and Figure 2), indicating that unmet needs were negatively associated with one’s own physical QOL. A significant actor effect of unmet needs was found on the MCS for both the cancer patients (β = −0.407, *p* < 0.001; Table 7 and Figure 2) and their families (β = −0.354, *p* < 0.001; Table 7 and Figure 2). However, no partner effects of unmet needs on QOL were shown for both the patients and their families, unlike the hypothetical model.

## 4. Discussion

This study examined the unmet needs of cancer patients and their families, as well as their QOL. Because patients and their family members have close interactions in the family system, the impact of their interdependence on the association between unmet needs and QOL needed to be investigated using appropriate dyadic analysis methods. Therefore, we tested the effects of unmet needs on the QOL of the dyads using the APIM model. The results showed that there were significant actor effects and insignificant partner effects. We will discuss the actor effects of the cancer patients and their families first, and then speculate on insignificant partner effects.

The cancer patients’ unmet needs and physical QOL were negatively correlated in this study. The results are consistent with those of Miniotti, et al. [23] studying on advanced colorectal cancer patients. Unlike the study by Miniotti et al., our study was not limited to colorectal cancer patients or advanced cancer stages. The majority of the patient participants were cancer patients in the early stages of their treatment, less than a year after the diagnosis. According to the study of Willems and colleagues [24] on cancer patients with various cancer types and time after treatment, there is heterogeneity of unmet needs based on time. Although our study and that of Miniotti and colleagues differed in the cancer characteristics of the participants, the association between unmet needs and QOL is consistent between the two studies. It is suggestive that unmet needs affect QOL, although unmet needs may change over time, treatment, and the patient’s condition. Therefore, regular evaluation and timely intervention by healthcare professionals are required to improve the QOL of cancer patients.

The cancer patients’ unmet needs were also negatively correlated with mental QOL in this study. Smith, et al. [25] reported that their unmet needs are more related to the mental QOL rather than the physical one. Our study demonstrated slightly higher standardized estimates in the MCS as compared to the PCS as well. Wen and colleagues [6] reported that symptom distress and functional limitation is profoundly related to the patient’s QOL. Thus, appropriate management of symptoms and functional ability along with psychological counseling programs are necessary for cancer patients. Because initial unmet needs are strongly related to the future unmet needs [26], assessing them and intervening in a prompt manner is also essential.

The unmet needs of the cancer patients’ families were significantly associated with the physical and mental QOL in our study, which is consistent with Doubova and Infante-Castaneda [27]. Some studies [16,28] measuring the overall QOL, without classifying them into physical and mental QOL, reported unmet needs of families to be negatively associated to QOL. Hodgkinson, et al. [29] indicated that the higher the unmet needs of the partners of cancer patients, the lower the MCS. Kim and Carver [30] compared the unmet needs and the QOL of the family at 2 and 5 years subsequent to the patients’ initial cancer diagnosis. Various unmet needs were found to be associated with the mental QOL at both points of time. In addition, the unmet needs at the earlier time point significantly predicted future mental QOL. Even if the current unmet needs of cancer patient’s family are not high, they have a significant impact on their mental QOL at a later point in time, suggesting that early intervention is required for their unmet needs from a long-term perspective.

In this study, although there were actor effects, no partner effects of unmet needs on QOL were detected. It is difficult to compare this result to that of the other studies due to the absence of an APIM research focusing on “unmet needs” as an independent variable and the QOL as a dependent variable thus far. However, there are some studies on patient–family dyadic effects of psychosocial variables on their QOL. Inconsistent results have been found regarding the effects of psychological factors such as loneliness and fear on the QOL. There were no partner effects in a dyadic study on loneliness and health-related quality of life in breast cancer patients and their informal caregivers [31]. However, in the study of Kim and colleagues, fear had different partner effects on physical or mental QOL. The cancer patients’ fear of recurrence did not have a partner effect on both physical and mental QOL of the caregivers, including family members and close friends [32]. Nevertheless, the families’ fear affected the physical QOL, not the mental QOL of the cancer patient. A study showed that there were no partner effects of stress on QOL, however, as a mediator between stress and QOL, family caregiver’s stress coping had a significant impact on patient’s QOL [33]. It is suggested that there may be moderators existing between unmet needs and QOL; hence, future studies are required.

Considering social support, Kelley, et al. [18] found no partner effect of perceived social support on the subjective health of colorectal patients and primary informal caregivers. However, in lung cancer patients, the impact of social support on subjective health at 5 months after diagnosis had an actor and not a partner effect, while only the partner effect was significant at 12-months after diagnosis. These results suggest that perceived social support has different partner effects depending on the cancer type and trajectory. Compared to perceived social support, social support as a coping strategy has a significant effect on QOL. A caregiver’s coping strategy based on social support has a partner effect on the patient’s QOL, whereas the patient’s coping strategy has no partner effect on the caregiver’s QOL [34]. Hamidou, et al. [35] reported that a cancer patient’s coping strategy based on social support has a partner effect on the caregiver’s physical QOL, while the caregiver’s coping strategy based on social support has a partner effect on the patient’s mental QOL. These findings indicate that there may be a mediation effect of coping strategies between social domains of unmet needs and QOL.

In this study, the highest rank of unmet needs for both the cancer patients and their families was related to health professionals and information. Our results are consistent with several studies showing that cancer patients and their family members require more information and supportive care from physicians and nurses [16,36,37]. It is suggested that additional intervention programs providing information and health professionals’ support are needed. In our study, the unmet needs did not differ based on the general characteristics, excluding the patients’ employment status. One of the reasons associated with lower unmet needs in employed patients is that working may provide an opportunity for receiving social support by interpersonal relationships at work, as well as financial benefits [38]. Furthermore, in this study, employment status was related to cancer patients’ physical and mental QOL, which is in line with previous studies [39,40]. Therefore, encouraging social activities, including working, is recommended for cancer patients if their physical functioning allows.

In addition to employment status, functional performance was found to be associated with physical and mental QOL of cancer patients in this study. It is suggested that patients with higher functioning have a better health condition that in turn is related to higher QOL. To maintain or improve their functional status, exercise interventions have been attempted [41]. Thus far, the programs seem to have affected the functional status positively, while the effects of exercise on QOL remain inconclusive. Future studies with robust research designs are needed to investigate these effects.

The physical QOL of family members was associated with age while the mental QOL was related to the family member’s employment and cohabitation with the patient. In this study, family participants who were 60 years or older showed a lower physical QOL. Previous studies on family members of terminal cancer patients also reported low QOL in the elderly or the patient’s spouse [27,42]. Living with a cancer patient was negatively related to the mental QOL of family members. This may be related to an increase in frequency of caring for patients and their psychological interactions with them. These results suggest that elderly family caregivers and cohabiting family members need additional supporting resources. Unemployed families demonstrated a lower mental QOL status. This finding is congruent with that of the previous studies showing that the socioeconomic status of cancer patients’ families had a significant effect on the QOL [43]. In addition, working may provide a form of social support. Therefore, cancer patients’ families are encouraged to engage in social activities, including work. In addition, communities need to expand their resources to support cancer patient care, which may ultimately improve cancer patients and their families.

This study has some limitations. First, the research design of this study was cross-sectional. A longitudinal study considering different time points in cancer trajectory should be considered. Second, the study was performed in an ethnic group. The interdependence between family members varies based on culture. The study should be repeated and extended to improve generalization or to understand regional differences. Third, it was not able to perform subgroup analysis according to cancer types or stages because the sample size of this study is relatively small. Further studies with large sample sizes are needed. Despite these limitations, our study has strengths, as it is the first to examine the impacts of unmet needs of cancer patients and their families on QOL, considering their interdependence as dyads using an actor–partner interdependence modeling approach. The results of this study show that there is a significant relationship between unmet needs and QOL, and both cancer patients and their families have high unmet needs for information and support from healthcare professionals. In conclusion, the unmet needs of both cancer patients and their families should be assessed and intervened in order to improve their QOL. It is also suggested that healthcare professions need to make more efforts to provide information and support to reduce unmet needs.

## Figures and Tables

**Table 1:** Characteristics of the participants (*N* = 115 dyads).

Variables		*n* (%) or Mean ± SD
Cancer patients	Gender	
(*n* = 115)	Male	65 (56.5)
	Female	50 (43.5)
	Age (in years)	61.90 ± 11.15
	Employment status	
	Employed	24 (20.9)
	Unemployed	91 (79.1)
	Cancer type	
	Lung cancer	7 (6.1)
	Gastric cancer	12 (10.4)
	Lymphoma	24 (20.9)
	Breast cancer	21 (18.3)
	Oral cavity, nasopharyngeal, esophageal cancer	9 (7.8)
	Gallbladder/biliary tract, hepatocellular carcinoma, pancreatic cancer	17 (14.8)
	Colorectal cancer	12 (10.4)
	Leukemia	4 (3.5)
	Bladder cancer, renal cell carcinoma	2 (1.8)
	Others (sarcoma, melanoma)	7 (6.0)
	Cancer period (in years)	1.57 ± 0.92
	<1	75 (65.2)
	1–3.9	24 (20.9)
	4–5.9	7 (6.1)
	≥6	9 (7.8)
	Current treatment	
	Chemotherapy	83 (72.2)
	Radiotherapy	2 (1.7)
	Chemotherapy & radiotherapy	9 (7.8)
	Supportive care	21 (18.3)
	Recurrence	
	No	93 (80.9)
	Yes	22 (19.1)
	Metastasis	
	No	18 (15.7)
	Yes	97 (84.3)
	Performance status (KPS)	73.48 ± 17.27
	≤40	6 (5.2)
	50–70	41 (35.7)
	80–100	68 (59.1)
Families	Gender	
(*n* = 115)	Male	30 (26.1)
	Female	85 (73.9)
	Age (in years)	50.94 ± 14.55
	Employment status	
	Employed	63 (54.8)
	Unemployed	52 (45.2)
	Caregiving period (in months)	
	<1	17 (14.8)
	1–5	42 (36.5)
	6–11	13 (11.3)
	12–35	21 (18.3)
	≥36	22 (19.1)
	Relationship with the patient	
	Mother	6 (5.2)
	Brother	3 (2.6)
	Sister	1 (0.9)
	Spouse	57 (49.6)
	Son	15 (13.0)
	Daughter	33 (28.7)
	Living with the patient	
	No	32 (27.8)
	Yes	83 (72.2)
	Alternative informal caregiver	
	No	48 (41.7)
	Yes	67 (58.3)

Abbreviations: KPS, Karnofsky Performance Status; SD, standard deviation.

**Table 2:** Descriptive statistics of the measured variables (*N* = 115 dyads).

Variables	Mean	±SD	Skewness	Kurtosis
Cancer patients’ unmet needs	50.93	±19.56	0.09	−0.52
Information	66.35	±21.81		
Psychological problems	59.01	±25.97		
Healthcare staff	66.99	±27.80		
Physical problems	49.64	±25.60		
Hospital facilities and services	57.68	±25.17		
Family/social support	40.97	±29.37		
Religious/spiritual support	28.84	±28.10		
Practical support	37.97	±25.12		
Families’ unmet needs	46.52	±20.68	0.19	−0.47
Health and psychological problems	41.06	±28.67		
Family/social support	33.04	±28.32		
Healthcare staff	67.83	±27.73		
Information	62.36	±24.73		
Religious/spiritual support	29.13	±31.60		
Hospital facilities and services	49.28	±26.64		
Practical support	37.78	±26.63		
Cancer patients’ PCS	38.06	±8.27		
Physical functioning (PF)	46.78	±27.87	−0.03	−1.07
Role physical (RP)	35.71	±28.16	0.56	−0.69
Bodily pain (BP)	43.56	±28.29	0.32	−0.72
General health (GH)	39.68	±19.07	0.03	−0.62
Families’ PCS	49.38	±7.68		
Physical functioning (PF)	75.96	±21.94	−0.79	−0.14
Role physical (RP)	71.63	±25.41	−0.72	−0.41
Bodily pain (BP)	67.05	±21.03	−0.23	−0.52
General health (GH)	54.09	±20.07	−0.10	−0.31
Cancer patients’ MCS	37.93	±11.24		
Vitality (VT)	38.59	±22.51	0.31	−0.57
Social functioning (SF)	50.22	±27.95	0.09	−0.89
Role emotional (RE)	43.55	±30.43	0.25	−0.98
Mental health (MH)	52.87	±22.63	−0.18	−0.49
Families’ MCS	42.13	±11.47		
Vitality (VT)	50.54	±21.15	−0.26	−0.89
Social functioning (SF)	67.07	±25.18	−0.65	−0.03
Role emotional (RE)	67.61	±27.08	−0.54	−0.62
Mental health (MH)	60.22	±22.04	−0.66	0.06

Abbreviations: MCS, Mental Component Summary; PCS, Physical Component Summary; SD, standard deviation.

**Table 3:** Differences in measured variables on the general and disease-related characteristics (Patient).

Characteristics	Unmet Needs		QOL			
			PCS		MCS	
	Mean ± SD	*t* or *F* (*p*)	Mean ± SD	*t* or *F* (*p*)	Mean ± SD	*t* or *F* (*p*)
Gender		−0.56 (0.579)		1.07 (0.288)		0.25 (0.806)
Male	50.04 ± 21.11		38.78 ± 8.79		38.16 ± 11.31	
Female	52.09 ± 17.49		37.12 ± 7.52		37.64 ± 11.24	
Age (in years)		2.26 (0.067)		1.17 (0.328)		0.90 (0.469)
<40	45.34 ± 20.22		30.78 ± 3.37		42.91 ± 17.05	
40–49	38.13 ± 19.33		40.19 ± 6.58		41.88 ± 12.40	
50–59	58.41 ± 18.36		38.53 ± 8.93		38.38 ± 10.72	
60–69	49.28 ± 20.33		38.86 ± 9.11		38.29 ± 11.81	
≥70	50.62 ± 17.71		36.34 ± 6.51		35.08 ± 9.88	
Employment		−3.15 (0.002)		2.86 (0.005)		2.12 (0.036)
Employed	40.17 ± 19.39		42.22 ± 6.52		42.20 ± 10.37	
Unemployed	53.77 ± 18.70		36.96 ± 8.36		36.81 ± 11.24	
Cancer type		1.29 (0.250)		1.11 (0.361)		1.02 (0.430)
Lung cancer	53.10 ± 22.83		34.98 ± 10.14		33.16 ± 14.49	
Gastric cancer	42.38 ± 24.28		40.42 ± 8.25		40.61 ± 10.87	
Lymphoma	44.03 ± 18.69		40.28 ± 8.06		41.18 ± 12.10	
Breast cancer	55.46 ± 18.21		37.02 ± 6.94		36.85 ± 11.25	
Oral cavity, nasopharyngeal, esophageal cancer	54.91 ± 16.44		37.74 ± 9.95		41.08 ± 10.31	
Gallbladder/biliary tract, hepatocellular carcinoma, pancreas cancer	59.68 ± 21.91		35.79 ± 8.67		33.04 ± 10.48	
Colorectal cancer	46.21 ± 19.83		41.12 ± 7.17		38.57 ± 9.80	
Leukemia	57.09 ± 10.33		31.43 ± 4.22		32.92 ± 5.98	
Bladder cancer, renal cell carcinoma	51.20 ± 13.92		34.34 ± 2.24		37.44 ± 17.23	
Others (sarcoma, melanoma)	51.64 ± 7.34		38.07 ± 10.60		40.03 ± 10.89	
Cancer period (in years)		1.25 (0.295)		0.53 (0.664)		0.13 (0.943)
<1	51.62 ± 20.34		38.63 ± 7.72		38.20 ± 11.76	
1–3.9	54.30 ± 18.07		37.78 ± 8.80		36.73 ± 9.46	
4–5.9	41.60 ± 15.99		35.98 ± 11.12		39.07 ± 12.85	
≥6	43.43 ± 17.78		35.63 ± 9.68		38.02 ± 11.57	
Current treatment		0.37 (0.774)		9.48 (<0.001) a, c > b		0.79 (0.500)
Chemotherapy ^a^	49.95 ± 19.53		40.00 ± 7.22		38.27 ± 11.48	
Radiotherapy ^b^	45.89 ± 26.34		26.32 ± 8.32		32.02 ± 5.18	
Chemotherapy & radiotherapy ^c^	52.57 ± 24.44		38.79 ± 9.64		41.47 ± 11.00	
Supportive care ^d^	54.58 ± 17.93		31.17 ± 7.48		35.65 ± 10.70	
Recurrence		0.94 (0.350)		−0.79 (0.429)		−0.79 (0.430)
No	51.76 ± 20.13		37.76 ± 8.27		37.53 ± 11.41	
Yes	47.41 ± 16.94		39.32 ± 8.34		39.65 ± 10.54	
Metastasis		−1.20 (0.230)		1.21 (0.229)		0.38 (0.708)
No	45.83 ± 19.83		40.22 ± 7.05		38.85 ± 12.62	
Yes	51.88 ± 19.47		37.66 ± 8.45		37.76 ± 11.03	
Performance status (KPS)		4.71 (0.011)		19.44 (<0.001) c > b > a		4.84 (0.010) c > a
≤40 ^a^	62.88 ± 13.20		27.38 ± 4.41		30.12 ± 6.98	
50–70 ^b^	56.42 ± 19.75		34.22 ± 7.99		34.97 ± 9.28	
80–100 ^c^	46.57 ± 18.84		41.31 ± 6.84		40.41 ± 11.95	

Note. a, b, c and d are labels designated for post-hoc comparison. See a, b, c located under the *p*-value in the right column. Abbreviations: KPS, Karnofsky Performance Status; MCS, Mental Component Summary; PCS, Physical Component Summary; QOL, Quality of life; SD, standard deviation.

**Table 4:** Differences in measured variables on the general and disease-related characteristics (Family).

Characteristics	Unmet Needs		QOL			
			PCS		MCS	
	Mean ± SD	*t* or *F* (*p*)	Mean ± SD	*t* or *F* (*p*)	Mean ± SD	*t* or *F* (*p*)
Gender		−1.42 (0.158)		1.75 (0.082)		0.91 (0.367)
Male	41.93 ± 19.92		51.47 ± 7.60		43.76 ± 11.75	
Female	48.14 ± 20.82		48.64 ± 7.62		41.55 ± 11.38	
Age (in years)		1.81 (0.132)		7.94 (<0.001) a, b > d, e		0.70 (0.596)
<40 ^a^	38.04 ± 20.02		54.68 ± 7.09		40.38 ± 10.83	
40–49 ^b^	47.25 ± 17.01		51.67 ± 6.42		44.92 ± 9.51	
50–59 ^c^	44.63 ± 18.92		48.53 ± 7.72		42.23 ± 13.66	
60–69 ^d^	52.38 ± 23.83		45.81 ± 6.65		41.77 ± 12.40	
≥70 ^e^	50.90 ± 21.44		43.29 ± 5.93		38.87 ± 7.94	
Employment		−0.4814 (0.629)		2.32 (0.022)		2.53 (0.013)
Employed	45.67 ± 20.54		50.86 ± 7.27		44.53 ± 10.91	
Unemployed	47.55 ± 21.01		47.58 ± 7.86		39.23 ± 11.55	
Duration of care (in months)		1.6910 (0.157)		1.74 (0.147)		0.18 (0.950)
<1	41.76 ± 20.04		52.36 ± 4.99		42.51 ± 7.62	
1–5	43.03 ± 19.10		50.22 ± 7.24		41.66 ± 12.22	
6–11	44.22 ± 24.13		47.24 ± 8.87		42.30 ± 12.36	
12–35	49.77 ± 19.43		49.40 ± 9.42		40.98 ± 13.87	
≥36	55.14 ± 21.84		46.69 ± 7.13		43.72 ± 10.13	
Alternative informal caregiver		1.0412 (0.300)		−4.37 (<0.001)		−0.41 (0.687)
No	48.90 ± 21.32		45.94 ± 7.21		41.76 ± 11.91	
Yes	44.82 ± 20.21		51.84 ± 7.09		42.64 ± 10.92	
Living together		−1.45 (0.151)		1.88 (0.063)		2.09 (0.039)
No	42.06 ± 16.86		51.52 ± 6.81		45.68 ± 10.06	
Yes	48.25 ± 21.83		48.55 ± 7.88		40.76 ± 11.73	

Note. a, b, c, d, and e are labels designated for post-hoc comparison. See a, b, c, d, e located under the *p*-value in the right column. Abbreviations: MCS, Mental Component Summary; PCS, Physical Component Summary; QOL, Quality of life; SD, standard deviation.

**Table 5:** Correlations among the measured variables.

Variables	X_1_	X_2_	X_3_	X_4_	X_5_	X_6_
Cancer patients Unmet needs (X1)	1					
PCS (X2)	−0.283 **	1				
MCS (X3)	−0.356 ***	0.312 **	1			
Families Unmet needs (X4)	0.385 ***	−0.240 **	−0.044			
PCS (X5)	−0.066	0.038	0.112	−0.241 **		
MCS (X6)	−0.232 *	0.255 **	0.249 **	−0.306 **	−0.028	1

Note. * *p* < 0.05, ** *p* < 0.01, *** *p* < 0.001.

**Table 6:** Actor–partner effects and the model fit (PCS).

	Exogenous Variables	Endogenous Variables		Standardized Estimates (*β*)	Standardized Error	*p*-Value	SMC
Actor effect	Cancer patients’ unmet needs	Cancer patients’ PCS		−0.40	0.11	<0.001	0.17
Partner effect	Families’ unmet needs			−0.04	0.09	0.680	
Actor effect	Families’ unmet needs	Families’ PCS		−0.40	0.10	<0.001	0.17
Partner effect	Cancer patients’ unmet needs			−0.02	0.10	0.850	
Model fit indices	*χ*^2^ (*p*)	*χ*^2^/*df*	GFI	TLI	CFI	RMSEA	SRMR
Reference	(>0.05)	≤3	≥0.9	≥0.9	≥0.9	<0.08	<0.08
Hypothetical model	38.38 (0.07)	1.42	0.94	0.94	0.96	0.06	0.06

Abbreviations: CFI, Comparative Normed of Fit Index; GFI, Goodness of Fit Index; NFI, Normed Fit Index; PCS, Physical Component Summary; RMSEA, Root Mean Square Error of Approximation; SMC, squared multiple correlation; SRMR, standard root mean squared residual; TLI, Tucker-Lewis Index.

**Table 7:** Actor–partner effects and the model fit (MCS).

	Exogenous Variables	Endogenous Variables		Standardized Estimates (*β*)	Standardized Error	*p*-Value	SMC
Actor effect	Cancer patients’ unmet needs	Cancer patients’ MCS		−0.41	0.10	<0.001	0.16
Partner effect	Families’ unmet needs			0.03	0.09	0.743	
Actor effect	Families’ unmet needs	Families’ MCS		−0.35	0.08	<0.001	0.18
Partner effect	Cancer patients’ unmet needs			−0.13	0.08	0.208	
Model fit indices	*χ*^2^ (*p*)	*χ*^2^/*df*	GFI	TLI	CFI	RMSEA	SRMR
Reference	(>0.05)	≤3	≥0.9	≥0.9	≥0.9	<0.08	<0.08
Hypothetical model	33.02 (0.20)	1.22	0.95	0.98	0.99	0.04	0.05

Abbreviations: CFI, Comparative Normed of Fit Index; GFI, Goodness of Fit Index; MCS, Mental Component Summary; NFI, Normed Fit Index; RMSEA, Root Mean Square Error of Approximation; SMC, squared multiple correlation; SRMR, standard root mean squared residual; TLI, Tucker-Lewis Index.

## Data Availability

The data presented in this study are available on request from the corresponding author. The data are not publicly available due to privacy issues.
